# Ear Acupressure for Smoking Cessation: A Randomised Controlled Trial

**DOI:** 10.1155/2013/637073

**Published:** 2013-09-28

**Authors:** Anthony L. Zhang, Yuan Ming Di, Christopher Worsnop, Brian H. May, Cliff Da Costa, Charlie C. L. Xue

**Affiliations:** ^1^School of Health Sciences, Traditional & Complementary Medicine Research Program, World Health Organization Collaborating Centre for Traditional Medicine, Health Innovations Research Institute, RMIT University, P.O. Box 71, Bundoora, VIC 3083, Australia; ^2^Department of Respiratory and Sleep Medicine, Austin Hospital, 145 Studley Road, Heidelberg, VIC 3081, Australia; ^3^School of Mathematics & Geospatial Sciences, RMIT University, P.O. Box 71, Bundoora, VIC 3083, Australia; ^4^Guangdong Provincial Academy of Chinese Medical Sciences, Guangzhou, China

## Abstract

This study investigated the efficacy and safety of ear acupressure (EAP) as a stand-alone intervention for smoking cessation and the feasibility of this study design. Adult smokers were randomised to receive EAP specific for smoking cessation (SSEAP) or a nonspecific EAP (NSEAP) intervention which is not typically used for smoking cessation. Participants received 8 weekly treatments and were requested to press the five pellets taped to one ear at least three times daily. Participants were followed up for three months. Primary outcome measures were a 7-day point-prevalence cessation rate confirmed by exhaled carbon monoxide and relief of nicotine withdrawal symptoms (NWS). Intention-to-treat analysis was applied. Forty-three adult smokers were randomly assigned to SSEAP (*n* = 20) or NSEAP (*n* = 23) groups. The dropout rate was high with 19 participants completing the treatments and 12 remaining at followup. One participant from the SSEAP group had confirmed cessation at week 8 and end of followup (5%), but there was no difference between groups for confirmed cessation or NWS. Adverse events were few and minor.

## 1. Introduction

Smoking is the largest single preventable cause of death and disease worldwide [1]. It is estimated that tobacco kills nearly six million people annually [2]. Despite quit-smoking campaigns and the availability of nicotine replacement therapy (NRT), quit attempts are often unsuccessful [3]. Nicotine withdrawal symptoms (NWS) including emotional distress, depressed mood, anxiety, and sleep disturbances present a barrier to successful smoking cessation attempts [4–6]. Increase in body weight associated with quitting smoking may also discourage some smokers [7].

Various forms of NRT are often combined with behavioural support to assist smoking cessation [8]. Other pharmacotherapies for smoking cessation include the antidepressant bupropion and the nicotine receptor partial agonist varenicline [9]. There is evidence for the efficacy of these therapies, but cessation rates remain relatively modest [10, 11], and the effects of interventions may last only a few months [12]. Additionally, NRT has been associated with increased adverse events (AEs) [13], and serious AEs have been associated with varenicline and bupropion [14, 15]. Since these pharmacotherapies do not help all smokers, there is a need to identify and evaluate other therapeutic options and new combinations of therapies.

Positive results have been reported for the use of ear acupuncture/acupressure for smoking cessation. One study (*n* = 141) compared a combination of true acupuncture and education versus sham acupuncture plus education and reported a higher cessation rate for true acupuncture which was still evident at a 3-month followup [16]. Wing et al. compared real ear-plus-body acupressure (*n* = 38) with sham ear plus body acupressure (*n* = 32) in active smokers for 3 weeks with a followup at 3 months. Both groups reduced smoking but there were no significant differences between groups [17]. In a study of ear acupuncture versus a sham acupuncture treatment for 8 weeks with followups for 6 months, Wu et al. reported no difference in cessation rate, but both groups reduced cigarette consumption and the EAP group (*n* = 59) showed a significant decrease in NWS [18]. Currently the evidence for the effect of EAP for smoking cessation is inconclusive and high quality trials with rigorous methodologies have been recommended [19, 20].

Trials of EAP have employed a variety of designs, acupuncture points, and methods of stimulation. In this randomized participant-and-assessor-blind clinical trial, noninvasive ear acupressure (EAP) was done on 5 points as recommended in the literature for smoking cessation and used in previous trials (see Protocol [21]). The control intervention was EAP on five points considered as not specific for smoking cessation. This control was selected because there are a large number of acupoints on the ear, so it is difficult to construct a control that uses nonacupoints on the ear that is sufficiently plausible to enable effective blinding. A similar method was used with success in an earlier study [22]. The study aimed to investigate the efficacy and safety of this EAP intervention for assisting smoking cessation and the management of NWS and to determine the feasibility of this study design.

## 2. Methods

The study was conducted at RMIT University, Australia, approved by the RMIT Human Research Ethics Committee, and registered with the Australian New Zealand Clinical Trials Registry (no. ACTRN12611000761965), and a protocol has been accepted for publication [21].

An independent researcher conducted block randomisation using a computer generated randomisation list. The randomised group codes were placed into sealed, opaque envelopes by another independent person. Participants, data entry personnel, and data analysts were blinded to treatment allocation. The single acupuncturist was trained to perform the same procedures at each consultation and was instructed not to discuss any aspect of the treatment procedures with the participants, to ensure participant blinding and consistency of treatment.

Participant recruitment was via local newspaper advertisements, posters, and the RMIT University website. Inclusion criteria are aged 18 years or over, smoked >10 cigarettes per day for one year or more, and intending to quit smoking on the agreed date. Exclusion criteria are pathological condition of the ear; wearing a hearing aid; allergy to adhesive dressings; currently using NRT, bupropion, or varenicline; being enrolled in another quit smoking program; other persons from the same household already having been accepted into this study; use of antidepressant or antipsychotic medications; alcohol or substance abuse; pregnancy; use of ear acupuncture or ear acupressure for a respiratory condition and/or smoking cessation within the last 12 months; being a student or practitioner of acupuncture, or not able to read or understand English.

The trial comprised a 2-week run-in period, 8 weeks of treatment, and 12 weeks of followup. At their first visit, participants were given a thorough explanation about the study procedure, including the 50/50 chance of being randomly assigned to either the smoking-specific treatment group or the nonspecific treatment group. Participants were informed that they could choose to use NRT during the trial under medical advice and that the use of NRT would not disqualify them from continuing. If they agreed to participate, they signed informed consent documents. Following the run-in period and before the first treatment, participants randomly selected a sealed opaque envelope with their randomisation code inside. This allocated them to the smoking-specific-ear acupressure (SSEAP) group or the nonspecific ear acupressure (NSEAP) group. Participants selected a quit date at the first treatment which had to be before the third treatment.

During the 8-week treatment, participants received either SSEAP or NSEAP once per week. All participants received stainless-steel press-pellet tapes (Magrain Pellets: Cat. no. PELSST S/Steel Tan, supplied by Acuneeds Co., Australia) on five EAP points. Each pellet was 1.2 mm in diameter and attached to a round adhesive tape (7 mm in diameter) in a tan colour. The skin was cleaned and sterilised with a 70% alcoholic swab. Then, pellets were taped onto one of the participants' ears with no skin penetration. Then, the acupuncturist pressed each pellet for 10 seconds or until the ear became reddish and/or slightly sore. Participants were instructed on how to press the pellets and were requested to press all pellets three times daily throughout the week and whenever they felt a craving for cigarettes. At the subsequent visit the remaining pellets were removed by the acupuncturist and new pellets were placed on the opposite ear. Consultations lasted approximately for 10 minutes.

In the SSEAP group the following EAP points were used: Shenmen (TF4), Fei “lung” (CO14), Kou “mouth” (CO1), Hunger (extra), and Gan “liver” (CO12) [23, 24]. The points used in the NSEAP group were Lun 2 “helix 2” (HX10), Jian “shoulder” (SF4, 5), Suogu “clavicle” (SF6), Zhen “occiput” (AT3), and Ya “tooth” (LO1). The NSEAP ear points are not typically used for smoking cessation or respiratory conditions [23, 24].

The Fagerstrom Test for Nicotine Dependence was administered at baseline to assess the equivalence of groups [25]. To assess participants' determination to quit smoking, the Contemplation Ladder was administered at baseline, end of treatment, and end of followup [26]. Self-report data on NWS, cigarette consumption, NRT usage, and AEs were collected using Case Report Forms (CRFs) which participants completed daily throughout the study period.

Primary outcome 1 was smoking cessation. Successful cessation was defined as a 7-day point prevalence (no smoking over 7 consecutive days) [27] measured by participant self-report and validated by two consecutive exhaled carbon monoxide (CO) concentration readings of ≤10 ppm using the piCO+ Smokerlyser (Bedfont Scientific Ltd., Upchurch, Kent, UK). Exhaled CO measurements were conducted at each visit and recorded in the CRF (visit 1–visit 10).

Primary outcome 2 was NWS assessed using the Mood and Physical Symptoms Scale (MPSS) in which participants rate the severity of depression, irritability, restlessness, hunger, poor concentration, anxiety, insomnia, frequency and levels of smoking urges, and physical symptoms such as mouth sores, constipation, and cough or sore throat [28]. All questions had scoring range of 1–5 (low to high) except for Questions  8 and  9 (frequency and levels of smoking urges) which scored 0–5.

Secondary outcome measures included (1) NRT usage throughout the trial; (2) daily consumption of cigarettes; (3) body weight (measured each time the participant was on site); and (4) quality of life using the Short-Form 36v2 Health Survey (1992, 2003 Health Assessment Lab, Medical Outcomes Trust and Quality Metric Incorporated, USA) at initial assessment, end of treatment, and end of followup periods.

All participants were asked to record in their CRFs any unpleasant experience throughout the trial and to assess whether the AE was related to the EAP. Participants answered a question on the CRF at the end of the first and the last treatment weeks regarding which group they perceived that they had been allocated into.

*Statistical Analysis.* Statistical Package for the Social Sciences software version 18.0 (SPSS Inc., Chicago, Ill, USA) was used for analyses. Quality Metric Health Outcomes Scoring Software 4.0 was used for SF36 scores (2010 Quality Metric Incorporated, USA). Intention-to-treat analysis was applied with missing data being managed using expectation maximisation. Baseline characteristics of the groups were assessed using unpaired *t*-tests and *χ* ^2^ tests for equivalence. For primary and secondary outcome measures, unpaired *t*-tests or general linear model (GLM) was used as appropriate to compare group differences. The randomisation code was broken after the analysis of outcome measures was completed.

## 3. Results

**Figure 1:**
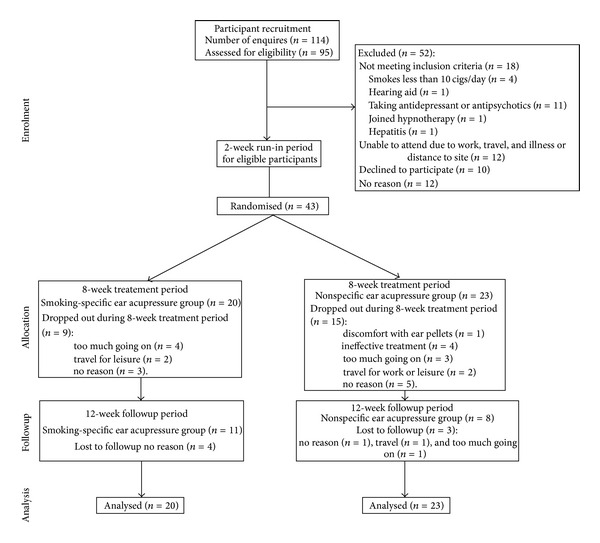
Participant recruitment and trial procedure.

Ninety-five (95) smokers were screened for eligibility for this study, eighteen did not meet inclusion criteria, 10 declined to participate, 12 were unable to participate due to various reasons (work, distance to trial site, overseas travel, and illness), and 12 participants were not contactable after the screening process (Figure 1).

Forty-three participants were randomised into the SSEAP group (*n* = 20) or the NSEAP group (*n* = 23). At the end of treatment week 8, 19 participants remained in the study and 12 remained till the end of followup. Thirteen dropouts gave no reason and were lost to contact. For those who gave a reason (*n* = 18), the most common was “too much going on” (*n* = 8) followed by 5 who dropped out because of travel. Four people in the NSEAP group reported that the treatment was ineffective, and one person in this group gave discomfort with ear pellets as the reason for dropping out (Figure 1).

At baseline, no significant differences between the two groups were found on gender, age, marital status, education level, and country of birth (Table 1). Comparison of participants' smoking history, presence of other household smokers, mean scores on Fagerstrom Test for Nicotine Dependence, mean scores for the Contemplation Ladder, exhaled CO, and body weight were not significantly different between groups. Nicotine dependence was medium to high in 86% of participants and all but two had made previous attempts to quit smoking. SF36 scores showed no significant differences between the groups for physical or mental components.

### 3.1. Smoking Cessation

During the treatment period, the CO-confirmed (10 ppm) 7-day-point prevalence cessation was three participants, one from the SSEAP group and two from the NSEAP group with no significant difference between groups (*χ* ^2^ = 0.225, *P* = 0.635). By the end of treatment, the two confirmed quitters in the NSEAP group had dropped out and were lost to followup. At three-month followup, the one participant from the SSEAP group had continued to abstain from smoking (CO confirmed), so the cessation rates were 5% for SSEAP and 0% for NSEAP at end of treatment and at end of followup. A post hoc analysis using a CO threshold of 7 ppm, which was at the lower end of the manufacturer's recommended range for the Smokerlyser, excluded one quitter from the NSEAP group but did not change the overall result.

### 3.2. Nicotine Withdrawal Symptoms

All MPSS scores were low at baseline. At baseline, there were significant differences between groups on the mean MPSS scores for Question  8 “frequency of urges to smoke” (*t* = −3.302, df = 41, and *P* = 0.002) and Question  9 “magnitude of urges to smoke” (*t* = −2.760, df = 41, *P* = 0.009). These two scores were used as covariates in a GLM analysis for these two questions to adjust to this difference. At the end of treatment and at the end of followup, there were no significant differences between the two groups for any of the NWS measured by MPSS.

### 3.3. NRT Usage

NRT usage was allowed, but only one participant from the SSEAP group reported NRT use. This participant did not quit or reduce cigarette consumption.

### 3.4. Cigarette Consumption

Based on participants' self-report, the mean daily cigarette consumption decreased from baseline to the end of treatment in both the SSEAP (15.14 ± 7.44, 10.61 ± 7.56) and NSEAP (19.11 ± 6.85, 12.32 ± 5.47) groups. The decreases were 30.0% and 35.5%, respectively. At the end of treatment, there was no significant difference between groups in cigarette consumption (*t* = −0.857, df = 41, *P* = 0.397). At the end of followup, consumption had increased compared with the end of treatment (SSEAP 14.5 ± 4.57 versus NSEAP 17.76 ± 5.03), but both groups remained lower than their respective baselines. A small but significant difference was found between groups (*t* = −2.203, df = 41, *P* = 0.033) at the end of followup.

### 3.5. Change in Body Weight

The mean individual change in body weight compared to baseline was not significantly different between groups at the end of treatment (SSEAP = 5.37 ± 9.38, NSEAP = −1.30 ± 14.09, *t* = 1.797, df = 41, *P* = 0.08) or at the end of followup (SSEAP = 6.80 ± 10.44, NSEAP = −0.17 ± 14.82, *t* = 1.754, df = 41, *P* = 0.087).

### 3.6. Quality of Life

There were no significant differences between the SSEAP and NSEAP groups on the physical (SSEAP = 49.30 ± 6.92, NSEAP = 49.41 ± 3.91, *t* = −0.07, df = 41, *P* = 0.945) or mental component scores (SSEAP = 48.83 ± 5.44, NSEAP = 48.05 ± 4.55, *t* = 0.472, df = 41, *P* = 0.612) of SF36v2 at end of treatment. Also, at the end of followup, no group differences were found on the physical (SSEAP = 49.27 ± 5.52, NSEAP = 47.97 ± 3.05, *t* = 0.967, df = 41, *P* = 0.339) or mental component scores (SSEAP = 55.01 ± 2.81, NSEAP = 53.77 ± 4.45, *t* = 1.071, df = 41, *P* = 0.29).

### 3.7. Credibility of Blinding

There was no significant difference between the two groups in participants' perception of group allocation at the end of the first treatment week (*χ* ^2^ = 5.035, df = 2, *P* = 0.081) or at the end of treatment week 8 (*χ* ^2^ = 3.753, df = 2, *P* = 0.153). Also, participant's belief regarding their group allocation did not significantly affect whether they dropped out or not.

### 3.8. Adverse Events (AEs)

All AEs (1 event from one SSEAP participant and 5 from four NSEAP participants) were mild or moderate, but one participant dropped out for discomfort with the ear pellets. The most frequent AEs were mild to moderate discomfort on the ear (1 in SSEAP and 4 in NSEAP). Participants reported that these AEs were “probably due to the EAP.” One participant in the NSEAP group reported slight headache and dizziness (1 event). All AEs were resolved without any medical intervention.

## 4. Discussion

This study compared the effects of an SSEAP intervention to an NSEAP intervention in adult smokers who wished to stop smoking. We employed the 7-day point-prevalence method to calculate cessation rate. One participant from the SSEAP group (5%) and 2 participants from the NSEAP group had quit smoking (8.7%) by the end of treatment. However, the only confirmed quitter at the end of treatment and at 3 months of followup was the SSEAP participant, since the two NSEAP participants did not continue in the study and all dropouts were considered as smokers in the ITT analysis.

Randomised trials of over-the-counter NRT patch versus placebo patch have reported successful quit rates of 5.6% to 15% in the treatment groups [3, 29–31]. Results from this study are at the lower end of this range. One possible reason for a low success rate in smoking trials is that these clinical trials tend to attract smokers who have already found it difficult to stop [32]. Notably, 95% of our study participants had previously tried to quit smoking and failed. Before trial commencement, we assessed participants' motivation to stop smoking using the Contemplation Ladder. Almost all participants (41/43) were determined to quit, but only 10 completed the study. In this study, high motivation to quit did not ensure success of quitting or indicate that the participant would complete the study.

High dropout rates in smoking cessation studies are not unusual [10]. Bier et al. in 2002 compared the effect on smoking cessation of EAP alone and in combination with education. During the 18-month trial period, the participants' numbers dropped from 141 to 48 [16]. Another study that compared the effect of NRT in combination with EAP had 7 out of 19 participants who completed the study [4]. Several factors may have contributed to the dropout rate in this study. Smokers committed to the study in good faith and aimed to quit smoking, but some reported that they stopped coming due to “too much happening in their lives,” or travel for work or leisure. Participants were required to visit the trial site weekly throughout the treatment period and were required to fill out the CRF daily. These demands on their time may have contributed to the high dropout rate, but we did not receive any feedback confirming this. We observed that participants frequently expressed how difficult it was to quit smoking and wished to discuss their experiences. However, due to the nature of acupuncture, the acupuncturist could not be blinded and therefore was instructed to have minimal interaction with participants. Therefore, participants may not have felt supported in their quit efforts. Counselling is widely available in Australia via helplines, and participants were advised to consult, their general practitioner, but future studies should include a form of support as part of the trial.

No differences in NWS were found between groups. The mean NWS scores were very low at baseline and throughout the trial. This could mean that the participants were not diligent in recording their NWS in the CRFs or that many participants did not make attempts to quit on their designated quit day. Post hoc investigation of the individual data found that the known quitters (*n* = 3) showed short-term increases in their scores for questions which measured cigarette cravings, whereas the known nonquitters showed little change. Therefore, change in the MPSS indicated the presence of individual cessation attempts at particular time points, so MPSS was found to be sensitive to changes in cigarette cravings. However, these changes were not detectable in the consolidated data because they lasted for short periods and occurred at different time points. In future trials a different approach to assessing NWS between groups should be considered.

There was an overall reduction in cigarette consumption. For those participants who completed the treatment period, in the SSEAP group (*n* = 11), one participant reduced his/her daily cigarette consumption by 75% compared to baseline, two participants reduced it by 47%, and one participant reduced it by 42%. In the NSEAP group (*n* = 8), one participant reduced his/her daily cigarette consumption by 32%, while another participant achieved a reduction rate of 15%. It is interesting to note that although most participants did not quit smoking, those receiving the SSEAP treatment showed a greater reduction in the number of cigarettes they smoked during the treatment period and a less pronounced rebound during followup. Cutting down is a step towards complete cessation [33]; therefore, EAP use may assist in reducing to quit. Similarly, a trial that compared active ear-plus-body acupressure with sham ear-plus-body acupressure in active smokers found that cigarette consumption was reduced [17].

Although one participant dropped out for discomfort with the ear pellets, AEs were all mild or moderate. Previous studies that employed a similar EAP protocol for persistent allergic rhinitis [22, 34] and smoking cessation [4, 17] found that EAP was well tolerated by participants.

*Issues with Study Design and Implementation.* Stainless-steel press-pellet tapes commonly used by Chinese medicine practitioners were used in the trial. Participants commented that the metal balls moved to a different spot after being pressed in between visits. Due to the small area of the ear and large number of acupuncture points, movement of the metal ball can mean that the participant has moved the pellet away from the ear point. This may have affected the results in the SSEAP group. White et al. in 2007 reported that ear pellets could become detached from the ear due to loss of adhesion [4]. Therefore, we took care to ensure that the skin was clean and pellet adhesion was good. The average weekly pellet loss was 17–24% which is approximately one pellet per week. While pellet loss was not a major issue, movement may have had an equivalent effect. Therefore, one week may be an unrealistically long time to expect the pellets to remain on the correct points, when the participants are repeatedly pressing them each day.

The dropout rate was a concern, and it affected the results since ITT analysis was used, and this required the imputation of a large number of data points based on conservative estimates. It was not possible to definitively determine the reasons for the high dropout rate, but the requirement to complete a series of questions on the CRF daily (two pages) could have been perceived as onerous, and participants may not have carefully considered their responses to questions. Therefore, reduction in the number of secondary outcome measures may assist in compliance and may also provide better accuracy in reporting. No support or counselling was provided during the trial, although this was available to participants via helplines or their GPs. This aimed to isolate the effect of the EAP and not to introduce an additional source of variation, but future studies should use a more pragmatic intervention that includes a form of support provided by an independent person blind to treatment allocation.

The ear points were selected based on existing literature and use in other clinical trials [21]. In general, the point “lung” is used for lung disorders, and “shenmen” is used to calm the mind. “Liver” is for liver dysfunction and stress, while “mouth” and “hunger” aim to reduce cravings. However, there is no single well-established EAP treatment for smoking, so the points selected mainly aimed to reduce NWS and stress. The nonspecific control points have a range of uses. For example, “shoulder” (Jian) is used for shoulder pain and shoulder tension, and “occiput” is used for neurological disorders and for sedation. Therefore, this NSEAP treatment cannot be considered a form of placebo, and some of these points could be indirectly beneficial for general stress and/or the relief of some symptoms associated with smoking cessation.

## 5. Conclusion

This study followed a rigorous methodological protocol, and blinding of participants was successful. Consistent with previous acupressure studies, only minor adverse events were reported. Dropout rates were high in both groups. ITT analysis found that after 8 weeks of treatment, the 7-day point prevalence, CO-confirmed quit rate in the SSEAP group was 5%, and this continued at three months after treatment, but there was no significant differences between groups. Cigarette consumption declined in both groups over the treatment period. The decline appeared more pronounced in the SSEAP group and there was a small statistical difference at followup. NRT usage was low throughout. There was no apparent effect on NWS in either group, but the method used appears unsuitable for detecting between group changes. Future studies should address the design issues found in this trial and investigate continuous abstinence and long-term cessation rate at 6 months.

## Figures and Tables

**Table 1:** Baseline data for smoking-specific and nonspecific ear acupressure groups.

	Smoking-specific ear acupressure group, *n* or mean ± SD (*N* = 20)	Nonspecific ear acupressure group, *n* or mean ± SD (*N* = 23)	*t*-test or Chi-square test results
Gender			
Male	8	10	*χ* ^2^ = 0.053, *P* = 0.818
Female	12	13	
Age (years)	50.4 ± 11.49	49.8 ± 8.53	*t* = 0.202, df = 41, *P* = 0.841
(range)	(29–69)	(27–68)	
Marital status			
Single	5	7	*χ* ^2^ = 2.446, *P* = 0.294
Married/partnered	13	16	
Separated	2	0	
Educational level			
High school	11	18	*χ* ^2^ = 3.408, *P* = 0.182
TAFE	4	1	
University or more	5	4	
Country of birth			
Australia	14	16	*χ* ^2^ = 0.001, *P* = 0.975
Outside Australia	6	7	
Age at the first cigarette	16.3 ± 3.66	16.35 ± 6.27	*t* = −0.303, df = 41, *P* = 0.976
No. of years smoking	34.05 ± 11.11	33.87 ± 9.07	*t* = 0.059, df = 41, *P* = 0.954
Previous attempts to quit			
Yes	20	21	*χ* ^2^ = 1.824, *P* = 0.177
No	0	2	
Other smoker(s) at home			
Yes	5	11	*χ* ^2^ = 2.386, *P* = 0.122
No	15	12	
Karl Fagerstrom Test for Nicotine Dependence Score			
Low	4	2	*χ* ^2^ = 2.522, *P* = 0.283
Medium	10	9	
High	6	12	
Contemplation Ladder	9.4 ± 1.35	9.17 ± 1.75	*t* = 0.469, df = 41, *P* = 0.642
Smoking rate/day (no. of cigs)	15.14 ± 7.44	19.11 ± 6.85	*t* = −1.189, df = 41, *P* = 0.076
Total no. of cigs smoked in one week	106 ± 52.11	132.3 ± 48.69	*t* = −1.170, df = 41, *P* = 0.095
CO measurement (ppm)	23.4 ± 9.02	24.65 ± 10.27	*t* = −0.422, df = 41, *P* = 0.675
Body weight (kg)	71.08 ± 13.47	77.99 ± 18.82	*t* = −1.345, df = 41, *P* = 0.186
SF36 scores			
Physical component	45.97 ± 9.87	44.39 ± 9.22	*t* = 0.540, df = 41, *P* = 0.592
Mental component	46.32 ± 12.07	46.88 ± 11.01	*t* = −0.161, df = 41, *P* = 0.873

## Abbreviations

AEs: Adverse events
CO: Carbon monoxide
CRF: Case report form
EAP: Ear acupressure
MPSS: Mood and physical symptoms scale
NRT: Nicotine replacement therapy
NSEAP: Non-specific ear acupressure
SF-36: Short-form health survey
SSEAP: Smoking-specific ear acupressure.
